# Conformance to schizophrenia treatment guidelines in North West-Bank, Palestine: focus on antipsychotic dosing and polytherapy

**DOI:** 10.1186/1471-244X-13-179

**Published:** 2013-07-01

**Authors:** Waleed M Sweileh, Jihad Bani Odeh, Sa’ed H Zyoud, Ansam F Sawalha, Manal S Ihbeasheh

**Affiliations:** 1 Division of Pharmacy, College of Medicine and Health Sciences, An-Najah National University, Nablus, Palestine

**Keywords:** Antipsychotics, Prescribing pattern, Palestine

## Abstract

**Background:**

Analysis of the prescribing patterns of antipsychotic drugs can improve therapeutic outcomes. The purpose of this study was to evaluate the prescribing pattern of antipsychotics and its conformance to international treatment guidelines.

**Methods:**

A cross sectional study at primary psychiatric centers was carried out. Patients’ medical files were used to obtain demographic, medication and clinical information. International guidelines for schizophrenia were used to create conformance indicators. All statistical analyses were conducted using Statistical Package for Social Sciences.

**Results:**

250 patients were included in this study. A total of 406 antipsychotic agents were used; 348 (85.7%) were first generation antipsychotics (FGA). The prevalence of antipsychotic combination was 50.4% (n=126). There was no significant difference in positive (p=0.3), negative (p=0.06) and psychopathology (p=0.5) scores of schizophrenia symptoms among patients on monotherapy versus those on antipsychotic combination. Furthermore, no significant difference was observed in the annual cost of antipsychotic monotherapy versus combination therapy. One hundred and five patients (42%) were using optimum dose of (300 – 600 mg CPZeq) while the remaining were using sub or supra therapeutic doses. Analysis showed that use of depot, use of anticholinergic agents and increasing amount of total CPZeq were significant factors associated with antipsychotic combination.

**Conclusions:**

This study indicated that antipsychotic prescribing was not in conformance with international guidelines with respect to maintenance dose and combination therapy. Type of antipsychotic treatment regimen, combination versus monotherapy, was not associated with better clinical or economic outcome.

## Background

Schizophrenia is a devastating mental illness that impairs mental and social functioning. The stigmatization of people who have a mental illness adds to difficulties in their daily life and increases the probability that those patients will be offered services that are of inferior quality to their needs. In the Arab world, mental health services are far away from optimum and there is a lack of reliable epidemiological psychiatric data which does not enable rational planning for future psychiatric services, education and research [1,2].

To improve psychiatric services, a large number of countries and organizations throughout the world have developed their own guidelines for the treatment of schizophrenia [3]. There are several major and well known guidelines/algorithms for the treatment of schizophrenia: the American Psychiatric Association (APA) Practice Guideline for the Treatment of Patients with Schizophrenia [4]; the Schizophrenia Patient Outcomes Research Team (PORT) treatment recommendations [5]; and the Texas Medication Algorithm Project (TMAP) antipsychotic algorithm for schizophrenia [6]. Additionally, recent studies such as the Clinical Antipsychotic Trials of Intervention Effectiveness (CATIE) [7], the Cost Utility of the Latest Antipsychotic Drugs in Schizophrenia Study (CUtLASS) [8], and their comparisons have shed more light on the National Institute for Health and Clinical Excellence (NICE) schizophrenia guideline from the United Kingdom [9]. Such guidelines generally acknowledge that there is a lack of evidence to support the routine use of combined antipsychotics except when switching from one antipsychotic to another or augmenting clozapine in treatment-resistant illness [6,9,10]. Furthermore, there is a consensus among most guidelines about the optimum maintenance dose for treatment of schizophrenia.

The need to analyze the prescribing patterns of antipsychotic drugs is not to find blame but to identify areas where regulatory policies can improve prescribing patterns and outcomes. As Palestinian legislators and policy makers seek to tighten medication wastage, a study of the prescribing pattern of antipsychotic drugs will serve as a baseline data to help policy makers to implement protocols that are safe and cost effective in Palestinian authority.

The current study draws a sample of patients with schizophrenia to examine the antipsychotic prescribing pattern and conformance of antipsychotic drug prescribing with international guidelines. Particularly, frequency of antipsychotic combination and maintenance dose will be examined. Furthermore, factors associated with antipsychotic combination, and the clinical and economic impact of antipsychotic combination will be investigated.

## Methods

A cross sectional study was conducted between August 2011 and February 2012. The Palestinian territories, where the study took place, comprise the West Bank, Gaza Strip and East Jerusalem. This study was carried out at the 4 governmental primary psychiatric healthcare centers located in Nablus, Tulkaram, Jenin and Qalqilia in northern West-Bank, Palestine.

The centers included in the study were those in Nablus, Jenin, Tulkaram, and Qalqilia. Patients who fulfilled the following criteria were invited to participate: 1) their age was above 16 years old, 2) they were diagnosed with schizophrenia as defined by Diagnostic and Statistical Manual of Mental Disorders (DSM-IV), 3) they had not been suffering from an acute attack of psychiatric illness during the past year, and 4) their drug regimen had not been changed in the past 6 months as evident from their medical files. Patients who had the following characteristics were excluded from the study: 1) newly diagnosed patients and 2) patients who were not on any antipsychotic medication.

A convenience, non-probability sampling method was used. To collect the sample, the researchers visited and stayed 4 weeks in each center and collected data from patients who met the inclusion criteria. In order to estimate with sufficient precision the prescribing pattern, particularly, antipsychotic combination, we hypothesized a worst-case scenario level of antipsychotic combination of about 70%, to be estimated with a 95% confidence interval of 15 percentage points. Therefore, we needed a sample size of at least 134 patients. The final sample collected during the study period was 250 clients. We developed data collection forms to cover all data items needed. The form covered the following areas: socio-demographic variables, psychiatric history, antipsychotic medication currently being used, and history of psychiatric hospitalization. Before commencing the study, the data collection form was modified by the researchers and the modified version was reviewed by experts to ensure content and construct validity. Data from the pre-test evaluation were not included in the final analysis. Focus group discussions were continuously held between research team to identify any problems in data collection or interpretation of definitions or application of study criteria.

Approval to perform the study was obtained from the Palestinian ministry of health, the college of Graduate Studies at An-Najah national University and the Institutional Review Boards (IRB) committee at An-Najah National University. Written consent was obtained from all participants.

### Tested variables

#### Chlorpromazine dose equivalencies (CPZeq)

The CPZeq is a measure of the relative antipsychotic potencies of neuroleptics. They are generally expressed as a ratio, relative to the arbitrary value of 1, which corresponds to the antipsychotic effects of chlorpromazine. The daily dose of antipsychotic medication prescribed to each patient was converted to milligram equivalents of chlorpromazine according to conversion factors derived from the literature [11,12]. Total CPZeq was constructed by calculating a total daily dose of each antipsychotic listed in the medical file. Then each converted antipsychotic-specific CPZeq amount is added to arrive at a total dose. A maintenance dose of total CPZeq in the range of 300 – 600 mg is considered optimum.

#### Antipsychotic combination

The operational definition of antipsychotic combination is the use of two or more antipsychotic drugs while that of monotherapy is the use of one antipsychotic drug. In this study, since no changes were made in the therapeutic regimen of all clients, then no clients were in the transitional period of changing or switching from one antipsychotic to another. Therefore, clients who were on combination therapy were actually on 2 or more medications and were not in a transitional phase of discontinuation of one drug and an initiation of another drug.

#### Positive and negative syndrome scale (PANSS)

The Positive and Negative Syndrome Scale (PANSS) which was published in 1987 [13,14] is widely used in the study of antipsychotic therapy. The PANSS consists of three sections: positive symptoms (7 items); negative symptoms (7 items); and general psychopathology (14 items). Each item has a score from 1 – 7 with the highest scores representing the highest severity. For each section, the total score was calculated by summing the scores for all items in that section. The interview with the clients to assess PANSS was carried out by the author. The author (mental health specialist) was trained on PANSS before the start of the study. Each interview took 30 – 35 minutes.

#### Conformance indicators

In order to construct the indicators of conformance, we reviewed several updated guidelines regarding recommendations for maintenance pharmacological treatment of schizophrenia [9,10]. We identified two major recommendations that could be evaluated using a cross-sectional medical chart review, and for which the required information was available in the medical records.

I. The first conformance indicator recommends that monotherapy is the preferred and evidence based choice while combination therapy lacks evidence and support. Our cross sectional methodology could not be used to provide information on whether patients were undergoing a trial of second alternative antipsychotic drug (switching) or not. However, we assumed that all patients were not undergoing switching because their medications have been constant since more than six months. Therefore, we drafted a conformance indicator that examined whether patients were receiving one or combination.

II. The second conformance indicator recommends that the maintenance dosage of antipsychotic medication be in the range of 300 – 600 mg CPZeq per day. Since all patients in the study sample had been ill for a long time and their medications had not been changed since the past six months, we assumed that most patients were on maintenance doses and created a conformance indicator that assessed whether the antipsychotic dosages prescribed for patients were between 300–600 mg CPZeq/day.

#### Cost of antipsychotic regimen

The total annual cost for antipsychotic treatment for each patient was calculated in United State Dollar (1 USD = 3.7 New Israeli Shekel (NIS) at the time of the study). The cost of each antipsychotic drug per dose per unit was obtained from the Ministry of Health (personal communication).

### Data analysis

Descriptive statistics for all study variables were computed. These descriptive statistics included frequencies and percentages for all categorical variables in addition to means, standard deviations and ranges for all normally distributed continuous variables while median and inter quartile range (Q1-Q3) for continuous variables that were not normally distributed. Variables were tested for normality using the Kolmogorov– Smirnov test. Non-parametric binomial *t*-test was used to test the difference in the prevalence of antipsychotic combination obtained in this study with those published elsewhere. Statistical significance for intergroup differences was assessed by the Student’s *t*-test for continuous variables. Univariate analysis and multiple logistic regression was used to find significant factors associated with antipsychotic combination.

All statistical analyses were conducted using Statistical Package for Social Sciences SPSS (PASW version 18.0; IBM, Somers, NY) statistical packages for Windows. The conventional 5 percent significance level was used throughout the study.

## Results

### General characteristics of the study sample

During the study period, 250 patients met the inclusion criteria and were included in the study. The study sample represents approximately one third of patients with schizophrenia attending the primary governmental psychiatric clinics in north Palestine. The largest clinic in terms of number of schizophrenic patients attending the clinic on regular basis is the one in Nablus with approximately 350 patients followed by the ones in Tulkaram, Jenin and Qalqilia with approximately 80 – 120 patients each. The fact that schizophrenia patients have to attend the psychiatric clinic at least once monthly to get their antipsychotic medications made all patients at equal chance of being seen and included in the study since the authors spent one month in each clinic. It is noteworthy, that most attendants to psychiatric primary healthcare centers in north Palestine were male patients who mostly visit the clinic in regular basis for evaluation and to obtain medications. Patients not included in the study were either unwilling to expose their illness to researchers or were in a hurry and did not want to participate. Aside from the above reasons, there is a small percentage of patients who does not usually show at the clinics regularly. For the 250 clients studied, medical files were complete for antipsychotic medications and their doses. However, clinical data pertaining to psychiatric episodes, psychiatric hospitalizations, co-morbid diseases and duration of psychiatric illness were frequently missing.

The study sample included 68 (27.2%) female and 182 (72.8%) male clients. The mean age of the clients was 41.9 ± 11.8 [95% CI: 40.5 – 43.4] years. No significant difference in age was found between male and female clients (40.3 ± 12.4 for females versus 42.5 ± 11.5 years for males; p=0.2). The median duration of illness was 15 (Q1 – Q3: 9 – 20) years. The median number of psychiatric hospitalization of the clients during their lifetime was 1 (Q1 – Q3: 0 – 2). Basic demographic and clinical characteristics of the clients are shown in Table 1.

**Table 1:** General characteristics of the study sample

**Variable**	***N *(%) or median (Q1–Q3) or mean ±SD**
**Gender**	
-Male	182 (73.8%)
-Female	68 (27.2%)
**Age (years)**	41.9± 11.8
**Age category**	
-Less than 30	43 (17.2%)
-30 – 40	76 (30.4%)
-40 – 50	80 (32%)
-> 50	51 (20%)
**Residence**	
-City	105(42%)
-Village/camp	145 (58%)
**Education**	
-School education or less	213 (85.2%)
-College education	37 (14.8%)
**Marital Status**	
-Married	138 (55.2%)
-Single/divorced	112 (44.8%)
**Smoker**	
-Yes	153 (61.2%)
-No	97 (38.8%)
**Occupation**	
-Not working	219 (87.6%)
-Working	31 (12.4%)
**Duration of psychiatric illness** (years)	15 (Q1 – Q3: 9 – 20)
- ≤ 10 years	89 (35.6%)
- > 10 years	161 (64.4%)
**Number of psychiatric hospitalization**	1 (Q1 – Q3: 0 – 2)

Abbreviations: *Q1–Q3* lower quartile–upper quartile, *SD* standard deviation.

### Medications used by the study sample

There were 406 prescriptions of antipsychotic drugs for the study sample with a mean of 1.6 ± 0.7 (95% CI: 1.5 – 1.7) antipsychotic drug per patient. The antipsychotics were mainly from FGA type (348, 85.7%) The most common antipsychotic medication used was chlorpromazine tablet (128; 31.5%), followed by fluphenazine IM depot injection (125; 30.8%), haloperidol tablet (74; 18.2%), clozapine tablet (35, 8.6%), olanzapine tablet (15, 3.7%), haloperidol decanoate (11, 2.7%), risperidone tablet (8, 2%), trifluperazine tablet (7, 1.7%), thioridazine tablet (1, 0.2%) and zuclopenthixol depot (2, 0.5%).

The total number of adjuvant medications used was 249 with an average of 1 ± 0.7 (95% CI: 0.9 – 1.1) medication per patient. The most common adjuvant medications used by the clients were: the anticholinergic/antiparkinsonian drug, trihexyphenidyl (177; 71.1%) followed by antidepressants (29; 11.7%) and mood stabilizers (26; 10.4%) and benzodiazepines (anxiolytics) (17; 6.8%).

### Conformance to prescribing indicators

#### Antipsychotic combination

One hundred and twenty four clients (49.6%) were using antipsychotic while the monotherapy while the remaining clients were using antipsychotic combination. The various antipsychotic combinations are shown in Table 2. The most common combination was “FGA + FGA” (78/250; 31.8%) followed by “FGA + FGA + FGA” (24/250; 9.6%) and “FGA + SGA” (17; 6.8%).

**Table 2:** Types of antipsychotic regimens used in the treatment of schizophrenia

**Category**	**Frequency (%)**
One SGA	32 (12.8)
SGA + SGA	1 (0.4)
SGA + SGA + FGA	1 (0.4)
SGA + FGA	17 (6.8)
SGA + FGA + FGA	4 (1.6%)
One FGA	92 (36.8)
FGA + FGA	78 (31.2)
FGA + FGA + FGA	24 (9.6)
Total	250 (100)

*Abbreviations*: *FGA* First Generation Antipsychotics, *SGA* Second Generation Antipsychotic.

Analysis of cost indicated that the average annual cost of antipsychotic medications was 229 ± 202 USD per client (minimum: 48.7; maximum: 1168 USD). The average annual cost in USD per client was greater but not significantly different between clients on monotherapy versus those on antipsychotic combination (217 ± 200.7 USD for monotherapy versus 240.6 ±203.5 USD for combination therapy; p=0.4).

One hundred and twenty two clients out of 250 included clients in the study agreed to undergo the PANSS test. Of the 122 clients, 32 (26.2%) were female and 90 (73.8%) were male patients. The mean age of the patients was 41.5 ± 10.3 [95% CI: 39.7 – 43.4; range: 16 – 66] years. No significant difference was observed in age or gender distribution between patients who did and those who did not undergo the PANSS test (p=0.8; p=0.9 respectively). For the 122 patients, the average number of antipsychotic medications used was 1.7 ± 0.7 (95% CI: 1.6 – 1.9), mean CPZeq dose was 455.3 ± 283.4 (95% CI: 404.5 – 506.1), and 65 (53.3%) were receiving antipsychotic combination. Again, no significant difference in antipsychotic medications was observed between those who did and those who did not undergo the PANSS test. For patients with monotherapy, the means ± SD of positive, negative and psychopathology symptoms score were 20.8 ± 3.1; 19.7 ± 3.8 and 44.1 ± 6.3 respectively. For patients with combination therapy, the means ± SD of positive, negative and psychopathology symptoms score were 21.5 ± 4; 21.2 ± 4.5 and 45.1 ± 7.8 respectively. There was no significant difference in positive (p=0.3), negative (p=0.06) and psychopathology symptoms (p=0.5) scores between clients on monotherapy and combination therapy.

#### Antipsychotic maintenance dose

The average CPZeq dose measured in mg for the 250 clients was 436.9 ± 257.6 (95% CI: 403 – 471). No significant difference was found in CPZeq dose between male and female clients (386.8 ± 247.7 mg for females versus 455.5 ± 283.7 mg; p=0.63).

Categorization of CPZeq dose showed that 88 (35.2%) clients were using sub-therapeutic doses (<300 mg CPZeq), 105 (42%) were using optimum dose (300 – 600 mg CPZeq) and 57 (22.8%) were using supra therapeutic doses (> 600 mg CPZeq). Only 7 (2.8%) clients were using supra-maximal dose (CPZeq>1000 mg). Clients on antipsychotic combination had a mean CPZeq of 614.3 ± 267.1 (95% CI: 567.2 – 661.4 mg) while those on monotherapy had a mean CPZeq of 256.6 ±127.5 (95% CI 233.9 – 279.3 mg) (p <0.01).

### Factors associated with prescribing antipsychotic combination

Univariate analysis for antipsychotic combinations is shown in Table 3. It showed that antipsychotic combination was a significant associated with the following variables: smoking (p=0.04), duration of psychiatric illness > 10 years (p=0.01), number of psychiatric hospitalization ≥ 2 (p=0.001), use of depot antipsychotic agents (p<0.01), use of anticholinergics (p<0.01), and use of high CPZeq dose (p<0.01). Table 4 shows the multivariate logistic regression analysis of factors related to antipsychotic combination. All included variables had a significant p-value in the univariate analysis between antipsychotic combination and monotherapy groups. Results showed that the following variables are significant predictors of antipsychotic combination: use of depot medications, use of anticholinergic agents and use of high CPZeq dose.

**Table 3:** Univariate analysis (binary logistic regression) for prescribing monotherapy versus combination therapy

**Variable**	**Reference category**	**β**	**p-value**	**Odds ratio with 95% CI**
Gender	Male	0.43	0.14	1.5 (0.88 – 2.7)
Age	Continuous variable	0.008	0.5	1.0 (1.0-1.03)
Education	School education	0.338	0.4	1.4 (0.7-2.8)
Marital status	Single	−0.2	0.4	0.8 (0.5-1.3)
Smoking	Not smoking	0.45	0.04	1.7 (1.02-2.9)
Occupation	Not working	0.055	0.9	1.1 (0.5-2.2)
Waist circumferences	Normal WC	−0.16	0.5	0.9 (0.52-1.4)
Duration of psychiatric illness	< 10 years	0.7	0.01	2 (1.2-3.4)
Number of hospitalization	< 2	1.02	0.001	2.8 (1.5-5.1)
Family history of DM	No family history	−0.16	0.53	0.9 (0.42-1.4)
Depot	No depot	2.0	< 0.001	7.4 (4.2-12.9)
Anticholinergic	No anticholinergics	1.9	<0.001	6.7 (3.5-12.8)
SGA	No SGA	−0.4	0.2	0.7 (0.4-1.2)
CPZeq	Continuous variable	−5.5	<0.001	1.01 (1.0 – 1.01)

*Abbreviations*: *CI* confidence interval, *β* coefficient of predictor variables, *DM* diabetes mellitus, *SGA* Second Generation Antipsychotic, *CPZeq* Chlorpromazine Dose Equivalencies.

**Table 4:** Multivariate logistic regression for variables significantly associated with prescribing antipsychotic combination

**Variable**	**B**	**p-value**	**Odds ratio with 95% CI**
Smoker	0.181	0.661	1.199 (0.534-2.692)
Duration of psychiatric illness > 10 years	−0.435-	0.327	0.647 (0.271-1.545)
Number of psychiatric hospitalization ≥ 2	0.614	0.229	1.849 (0.679-5.034)
Use of depot antipsychotic agents	1.270	0.002	3.560 (1.572-8.061)
Use of anticholinergic agents	1.336	0.005	3.804 (1.511-9.578)
Total CPZeq	0.010	<0.001	1.010 (1.007-1.013)

Abbreviations: *CI* confidence interval, *β* coefficient of predictor variables, *CPZeq* Chlorpromazine Dose Equivalencies.

## Discussion

This study investigated conformance with antipsychotic treatment guidelines among schizophrenic patients attending governmental primary psychiatric healthcare centers in northern West-Bank of Palestine. We developed 2 indicators to measure conformance and appropriateness of antipsychotic prescribing with treatment guidelines for patients with schizophrenia: (1) the antipsychotic monotherapy and (2) antipsychotic maintenance dosing. Our study showed that antipsychotic prescribing was not in conformance with international guidelines with respect to maintenance dose and combination therapy.

In our study, the prevalence of antipsychotic drug combination was significantly higher than those reported in many other countries. A study in South Africa reported a prevalence of 28.6% of antipsychotic polypharmacy while a study in Nigeria reported a prevalence of 92% of antipsychotic combination [15,16]. Studies in USA and Canada reported prevalence of antipsychotic combination similar to that in South Africa (27.5% and 25.7% respectively) [17,18]. Reports in Asia also showed low (< 31%) prevalence of antipsychotic combination [19,20]. Discrepancies in prevalence of antipsychotic combination across studies may be accounted for by differences in the definition of antipsychotic combination. Although most studies defined combination as any time with more than one antipsychotic [21,22], others have set specific time requirements, such as at least 14 days of concurrent antipsychotic use [23]. Other potential reasons for variations in the prevalence of antipsychotic combination across studies include availability and type of medical insurance for schizophrenia patients as well as clinical experience and knowledge of psychopharmacology by medical practitioners [24].

Our results showed that antipsychotic combination was not associated with better clinical outcome. Similar findings were obtained by other studies. A study looking at combination of FGA and SGA found that there was little evidence of an improvement in outcome and there was a significant increase in adverse effect burden [25]. Another study found that use of combination antipsychotic agents was associated with an increased incidence of metabolic syndrome [26]. Honer et al. (2006) observed that antipsychotic polypharmacy (APP) led to greater worsening on verbal memory compared to antipsychotic monotherapy (APM) [27]. A double blind randomized control trial found no significant change in symptoms observed with APM augmentation on Brief Psychiatric Rating Scale (BPRS) [28]. On the contrary, an observational study of amisulpride added to clozapine for psychosis unresponsive to clozapine monotherapy observed that clozapine combination led to greater improvement in symptoms on BPRS [29].

In our study, antipsychotic combination was not significantly associated with patient’s gender. This is contrary to results reported in other studies that showed an increase in antipsychotic combination among male patients [23,30]. Equivocally, Chalks et al. (2006) reported an increase in antipsychotic combination among female patients [31]. Our finding regarding the association between anti cholinergic agents use and antipsychotic combination is consistent with previous studies [22,32,33]. Antipsychotic combination is expected to be associated with higher CPZeq effects which explains the significant association between antipsychotic combination and use of anticholinergic agents [22].

Our finding concerning the association of antipsychotic combination with depot antipsychotic medication was also proofed by other studies [12,22]. Treatment guidelines do not have particular recommendations regarding use of depot antipsychotic medications. It could be argued that due to limited mental health staff and services in many primary healthcare clinics in Palestine, physician and patients’ families prefer depot medications to minimize visits and avoid the daily burden on these clinics.

In our study, only 43% of patients received the recommended antipsychotic maintenance dose of 300–600 mg CPZ equivalents, while 26% received doses below the recommended doses and 32% received above the recommended doses. Similar results were reported in other studies [34,35]. Kreyenbuhl et al., (2007) observed that dosages prescribed for patients receiving polypharmacy were the same or modestly higher than those prescribed for patients receiving monotherapy [30]. Another study observed that CPZeq dose of patients on APP was significantly higher than those on APM which was in accordance with our findings [36]. [37] Also Barnes TR 2011 found that polypharmacy is a major contributor to high dose prescribing [37].

### Strength and limitations of the study

To the best of author‘s’ knowledge, this study is considered the first of its type and one of the few about mental health practices in Palestine. Several healthcare centers were included in the study and a relatively large sample size was obtained. However, our study has some limitations. First, the Palestinian culture and lack of psychiatric hospitals in north Palestine has affected some characteristics of the study sample. For example, female patients were under-represented in the sample possibly because families of female psychiatric patients seek private health services rather than governmental public healthcare services. Second, there were missing information in the medical file for some patients regarding psychiatric hospitalization, duration of psychiatric illness and co-morbid diseases. The sub-sample which was tested for the PANSS test is believed to be representative of the total sample since no significant difference in demographic and medication characteristics was observed between the sub-sample and the total sample. Third, the cross sectional design of the study limits interpretation of the results. The researchers stayed in each clinic for one month to make sure that all psychiatric clients have equal chance of being interviewed and included in the study. Despite this, the convenience sampling is a limitation and might possibly affect the representative nature of the sample. Fourth, most variables used in this study relied on information obtained from medical files. Finally, lack of a side effect assessment of study participants is also a limitation.

## Conclusion

Although, this study showed that antipsychotic combination is common, further studies are required to investigate the real intentions of the physician and whether prescribing of the combination is preceded by failure trails of monotherapy. Furthermore, more research is required to characterize the benefits and risks of antipsychotic combinations using different clinical tools for psychiatric assessment.

## Competing interests

The authors declared that they have no competing interests.

## Authors’ contributions

All authors read and approved the final manuscript. WMS: Concept, idea, literature review and manuscript writing. JBO: data collection. SHZ: Statistical analysis. AFS: data interpretation and manuscript writing. MSI: Data analysis, literature review, and manuscript writing.

## Pre-publication history

The pre-publication history for this paper can be accessed here:

http://www.biomedcentral.com/1471-244X/13/179/prepub
